# CACNA1C Polymorphism (rs2283291) Is Associated with Schizophrenia in Chinese Males: A Case-Control Study

**DOI:** 10.1155/2019/8062397

**Published:** 2019-04-01

**Authors:** Xiaojing Zhu, Rixin Li, Guojun Kang, Qi Kang, Wenwang Rao, Mingjia Yang, Bonan Cao, Mingyuan Zhang, Yaoyao Sun, Yueying Wang, Xin Chen, Yaqin Yu, Qiong Yu

**Affiliations:** Department of Epidemiology and Biostatistics, School of Public Health, Jilin University, Changchun 130021, China

## Abstract

Recent research has shown that prenatal famine exposure may be one of the risk factors for schizophrenia and that people born in famine years may be at an increased risk of schizophrenia due to alteration of the DNA methylation of genes. In this study, the association of rs2283291/rs4648635 and the incidence of schizophrenia and prenatal famine exposure at the genetic level were investigated to provide clues to the pathogenesis of schizophrenia. A total of 960 participants were recruited, comprising 473 prenatal famine-exposed individuals (225 patients and 248 controls) and 487 prenatal non-famine-exposed individuals (220 patients and 267 controls). The association of prenatal famine, schizophrenia, and their interaction with DNA methylation levels was analyzed using SPSS and GMDR software. Gender stratification analysis revealed a significant association between the rs2283291 genotype and schizophrenia in male patients (*P* = 0.017), and difference still existed after correction by the Bonferroni method. It was also found that an increasing risk of schizophrenia was associated with rs2283291 in males (OR: 1.62, 95% CI: 1.13-2.33, *P* = 0.0086, AIC = 669.7) in an overdominant model. The results of gene-environment interaction and gene-gene interaction revealed no association with the risk of schizophrenia. This study reported for the first time that rs2283291 was associated with schizophrenia in Chinese males.

## 1. Introduction

Schizophrenia (SCZ) is a serious mental illness with a lifetime prevalence of approximately 1%, with symptoms of affective disorder, cognitive disorder, and volitional behavior disorder [1], and its etiology is complex and involves genetic and environmental factors. DNA methylation plays a vital role in SCZ and can directly act as a pathogenesis or biomarker of SCZ [2]. Wilkinson [3] used genome-wide analysis in white blood cells, and global methylation results showed lower levels of DNA methylation in SCZ patients; thus, DNA methylation with the ability to regulate gene expression has shown its relationship with SCZ [4].

China experienced a serious three-year natural disaster from 1959 to 1961 (also known as the “famine” period), during which food was scarce and people were severely malnourished. Exposure to famine during pregnancy has a serious impact on the development of the foetus, and maternal protein deficiency especially methionine and folic acid deficiency can cause DNA methylation changes [5, 6]; folic acid is a key component of DNA methylation [7], and insufficient dietary supplementation of methionine, folic acid, vitamin B6, etc., can alter DNA methylation, thereby changing an offspring's phenotype. Retrospective studies have revealed that a poor utero environment caused by famine during pregnancy can lead to differences in DNA methylation in offspring [8]. Thus, people born during famine years may have altered DNA methylation due to nutrient deficiency.

Xu et al. [5] explained that exposure to famine during pregnancy has been identified as a risk factor for SCZ. Evidence from Dutch winter hunger from 1944-1945 and the 1959-1961 famine period in China shows that people exposed to famine during the foetal period especially in the first three months had a twofold increased risk of SCZ in adult life [9]. Harmful environmental events (like famine and nutritional deficits)—possibly through epigenetic mechanisms—may lead to SCZ [10]. DNA methylation is an inheritable epigenetic modification that can alter gene expression [11]; as a result, people born in the famine years may have an increased risk of SCZ due to altered DNA methylation of genes. This study explores the association between DNA methylation-related sites (rs2283291/rs4648635) and the incidence of SCZ as well as the effect of famine exposure at the genetic level, thus providing clues to reveal the pathogenesis of SCZ.

## 2. Materials and Methods

### 2.1. Subjects

Between 2010 and 2012, 960 SCZ patients and healthy people born in the famine years (1960-1962) and nonfamine years (1963-1965) in Jilin Province in the northeast of China were recruited. Exposure to famine during the prenatal period was defined based on birthdate, and the subjects were then divided into four groups: famine and SCZ group, famine and healthy control group, nonfamine and healthy control group, and nonfamine and SCZ group. A total of 445 SCZ patients were recruited from the Sixth Hospital of Changchun City and Siping Psychiatric Hospital. At least two independent experienced psychiatrists diagnosed the patients according to the tenth edition of International Classification of Diseases diagnostic criteria (ICD-10) [12]. Birthdate- and gender- matched control subjects were recruited from the Changchun Municipal Center for Disease Control and Prevention, and 515 healthy controls were free from mental illness. This study was approved by the Ethics Committee of the School of Public Health in Jilin University (approval number: 2014-05-01), and written informed consent was obtained from all subjects.

### 2.2. Genomic DNA Extraction and SNP Genotyping

Genomic DNA was extracted using Column Blood Clot DNA out (Win Honor Bioscience) and identified using a microplate nucleic acid protein analyzer (BioTek, USA), according to OD_260_/OD_280_ values to determine DNA content and purity.

SNPs were selected from an article “Mapping DNA methylation across development, genotype and schizophrenia in the human frontal cortex” [13] published in Nature Neuroscience based on MAF > 0.1. Then, according to the feasibility and applicability of the detection method, we selected only part of SNPs that can be detected. Furthermore, it has been found that *CACNA1C* is often considered a susceptibility gene for SCZ, while *PLCH2* has been reported to be associated with mental retardation. Therefore, two SNPs including rs2283291 (in intron 12 of *CACNA1C*) and rs4648635 (in intron 1 of *PLCH2*) were selected for detection.

SNP genotyping was carried out via the im LDRTM multiple SNP typing technology (Shanghai Tian Hao Biological Technology Co. Ltd. Genetic Analysis Center). Primer sequences of each SNP were as follows: rs2283291-F: GTGTTTGGCCCCGAGATGTT and rs2283291-R: GTTGCCAACTCAGGCTTGGA and rs4648635-F: CTTAGAGCCCCAGAACCGAG and rs4648635-R: GCAAGGGATGCCCCTCTAAG. The main steps of genotyping were as follows. (1) The region where the SNPs are located was firstly amplified by a multiplex PCR reaction in one system. (2) The amplified products were subjected to exonuclease and shinkinase (ExoI/SAP) purification for subsequent ligase reaction. (3) In a ligation reaction, each site contains two 5′ end allele-specific probes (the 3′ ends are two allele-specific bases or sequences, respectively, for insertion of a deletion polymorphism) as well as a fluorescently labelled specific probe next to a 3′ end site. The ligation product was differentiated by capillary electrophoresis of ABI 3730XL, and the original data file was analyzed using GeneMapper 4.1 software.

### 2.3. Statistics

Statistical analysis was performed using SPSS 24.0 and GMDR 0.9 software. Continuous variables were expressed as mean ± SD. Categorical variables were expressed as *N* (%). The chi-squared test was carried out to establish whether the genotype frequency distribution of SNPs was consistent with Hardy-Weinberg equilibrium (HWE), the genotype distributions and allele frequency distributions were compared using the chi-squared test or Fisher's exact test, and Bonferroni-adjusted*P* = 0.05/2 = 0.025was used as the critical value. SNP Stats online genetic analysis software [14] was used for genetic model analysis, with the smallest value of the Akaike Information Criterion (AIC) as the optimal genetic model. The gene-environment interactions were analyzed by logistic regression analysis while the gene-by-gene interactions were analyzed using the GMDR 0.9 software [15].

## 3. Results

### 3.1. Descriptive Analysis

Table 1 shows the baseline demographic characteristics between the four groups. The average age was 46.70-49.77 years, while the patients consisted of more males than females and more rural area people than urban area people. Maternal smoking, maternal drinking, maternal illness, and education level were similar among the four groups.

### 3.2. The Hardy-Weinberg Equilibrium Analysis

The genotype distributions of the two SNPs in both SCZ and healthy control groups satisfied the Hardy-Weinberg equilibrium (all *P* > 0.05) except for rs2283291 in the SCZ group (*P* = 0.036). About 10% of the samples randomly selected from the SCZ group were examined, and the accuracy of genotyping was more than 99%.

### 3.3. The Allele and Genotype Analysis

There was a significant association between rs2283291 genotype and SCZ in male patients (*χ*^2^ = 8.151, *P* = 0.017), and difference still existed after correction using the Bonferroni method. There was no association in females (*P* > 0.05), while rs4648635 and SCZ had no significant association for both males and females (all *P* > 0.05). Among the male subjects, the rs2283291 GA genotype of SCZ patients was significantly higher, while the frequencies of the GG and AA genotype were relatively lower, and there was no such difference among the female subjects. There was no significant difference in genotype distributions and allele frequencies of the rs2283291/rs4648635 between SCZ patients and healthy control groups or between famine and nonfamine groups (all *P* > 0.05). The associations between two SNPs and SCZ by famine exposure and gender stratification were further analyzed, and the results are presented in Tables 2 and 3.

### 3.4. The Inheritance Model Analysis

As shown in Table 4, the differences between SCZ and rs2283291 in males were statistically significant. According to the AIC values, the best genetic model was overdominant inheritance for an rs2283291 locus in the male subjects. In the overdominant model (AA+GG vs. GA genotype), it was found that the high risk of SCZ was associated with rs2283291 in males (OR: 1.62, 95% CI: 1.13-2.33; *P* = 0.0086, AIC = 669.7), but no difference was found in female subjects. The differences between SCZ and rs4648635 in both male and female were not statistically significant (all *P* > 0.05). Since there was no significant difference in genotype distributions of the rs2283291/rs4648635 loci between SCZ and healthy control groups (all *P* > 0.05), the optimal inheritance model of the association between SCZ and SNPs was further analyzed by gender stratification.

### 3.5. The Gene-Environment Interaction Analysis

As shown in Table 5, crossover analysis results based on the logistic regression analysis indicated that the interactions between the genotype in the rs2283291/rs4648635 loci and famine risk factor were not statistically significant (*P* > 0.05).

### 3.6. The Gene-by-Gene Interaction Analysis

**Figure 1:**
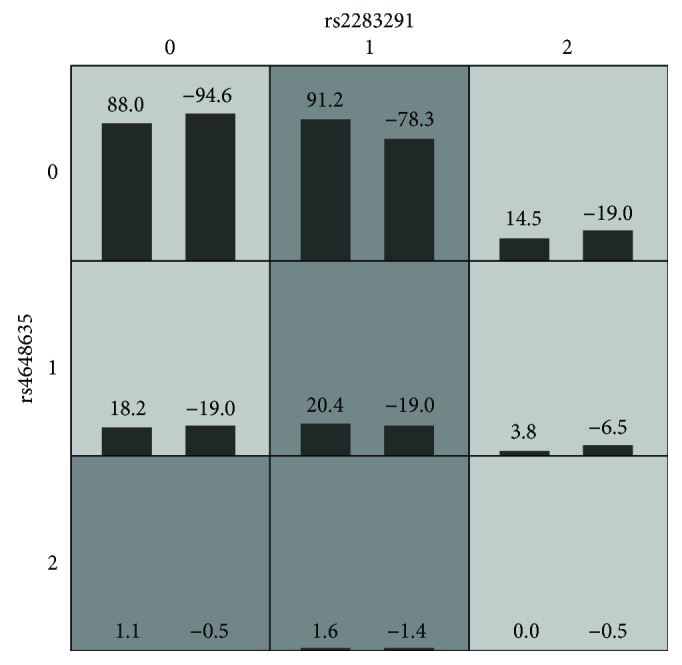
GMDR 2D interaction model in rs2283291 and rs4648635. The left bar represents the positive score and the right bar represents the negative score, and high risks are represented by dark shading and low risks by light ones. 0, 1, and 2 in the figure are the GG, GA, AA genotypes of rs2283291 and the CC, CT, TT genotypes of rs4648635, respectively.

In this study, the data from rs2283291 and rs4648635 were imported into GMDR (generalized multifactor dimension reduction) for analysis.  Figure 1 shows that in the SCZ group and the healthy control group, rs2283291 locus had a significant predominant effect and that the multifactor model 2 (rs2283291/rs4648635) crossvalidation consistency (CVC) was 10/10 and the training balanced accuracy rate was 53.28%, while the testing balanced accuracy rate was 51.04%. However, the constructed interaction multifactorial model 2 was not found to be associated with the risk of SCZ (*P* = 0.6230).

## 4. Discussion

Genome-wide association studies (GWAS) have reported that SNP rs2283291 and SNP rs4648635 loci are sites for susceptibility to SCZ [16]. Furthermore, Jaffe et al. [13] showed that rs2283291 and rs4648635 sites are significantly associated with methylation. Exposure to famine is a risk factor for SCZ and one of the factors contributing to methylation. Although this study did not find the two SNPs to be related to famine exposure based on the sample used, it reports for the first time that the methylation site rs2283291 was associated with SCZ in Chinese males. The results indicated that methylation may be potentially associated with SCZ in a Chinese population. Furthermore, the results showed that the GA genotype of the methylation site rs2283291 increased the risk of SCZ in the overdominant model. Overdominance, or an advantage of a heterozygote over both homozygotes, is usually adapted to the new optimal phenotype. The phenotype of the heterozygote is more prominent in the context of overdominance [17]. In addition, the rs2283291 was found to be a methylation-related site; thus, the heterozygous GA of rs2283291 reflects the methylation level which is the most dominant. Some reports indicate that heterozygotes can cause changes in the methylation level of SCZ; for example, Zong et al. [18] reported a significant association between the genotypes of SNP and the promoter DNA methylation (5mC) levels, and the heterozygous genotype (CT) of rs3811997 was correlated with the decreased 5mC levels in SCZ patients.

SCZ is a complicated hereditary disease, and its etiology is still unknown [19, 20]. Epigenetics is considered to account for the gaps in SCZ etiology research. This is because epigenetic modifications influence the development of organisms, especially in the embryonic and postnatal neural development and brain function. Increasing evidence shows that epigenetic changes are involved in the pathophysiology of SCZ. DNA methylation is one of the most important epigenetic modifications which regulate gene expression. It participates in neural development, and hence it may be a vital factor in the pathogenesis of brain diseases [4, 11, 20]. Through their studies using mouse models, Zhang et al. [21] concluded that the *CACNA1C* gene was related to SCZ and is a risk gene for many mental disorders such as SCZ and depression. *CACNA1C* encodes L-type voltage-dependent calcium channel Cav1.2 alpha-1c which modulates the permeability of the cell membrane to calcium ion, leading to intracellular signal transduction, gene transcription, and synaptic plasticity change, and this plays a vital role in the adjustment of the brains' major complex functions such as cognition, emotion, and behavior. The current study confirmed that there was a significant association between rs2283291 in the *CACNA1C* gene and SCZ. A significant difference was found in genotype frequencies of the *CACNA1C* rs2283291 among male SCZ patients, but female patients did not exhibit a significant correlation. Strohmaier et al. [22] suggested that *CACNA1C* has a distinct sex-specific effect and that it was involved in the internal phenotypic genetic structure of affective disorders and SCZ. In males, the A allele of rs1006737 within *CACNA1C* was associated with lower resilience and higher emotional lability, but the A allele was associated with stronger resilience and lower emotional lability in females. Although it is not the same SNP, their results showed a link to the gender difference in *CACNA1C*; it is therefore believed that *CACNA1C* may be different in male and female phenotypes. *CACNA1C* rs2283291 was examined in a case-control analysis, and it was found that the GA genotypes increase the risk of SCZ in males (OR: 1.62, 95% CI: 1.13-2.33, *P* = 0.0086). In addition to genetic factors, some reports suggest that gender differences in SCZ may be due to hormonal differences, the disease itself, and differences in other behavioral patterns. It is considered that the sex hormone oestradiol is used to induce a stabilizer-like effect on psychotic symptoms and has a neuroprotective effects on females [23, 24]. The most important reason is the difference in social baseline levels, with higher incidence of SCZ in males than in females. Moreover, before suffering from SCZ, females have fewer adverse social behaviors than males because of their late-onset age, higher education, work experience, and social ability, which facilitate their self-regulation and higher treatment compliance after the onset of psychiatric symptoms, and it is also a fact that females are able to cope better with the disease [25, 26].

Similar results from the Netherlands and Chinese famine investigation have proven that famine-induced maternal folic acid deficiency is a risk factor for SCZ [7]. Folic acid is essential for normal DNA methylation. Since humans cannot synthesize it, they usually obtain it from diet. Its deficiency impedes the generation of methyl donors and DNA methylation, which affects adjustment of gene expression associated with neurodevelopmental processes; low levels of maternal folic acid may influence the risk of offspring SCZ [27, 28]. The results in this study did not find any association between prenatal famine exposure and DNA methylation sites (rs2283291/rs4648635). Tobi et al. [29] suggested that prenatal famine exposure may cause changes in DNA methylation. There is a need to further explore the relationship between famine exposure and DNA methylation.

This study had some limitations. One was to reflect the level of methylation through methylation-related loci. Since the results in this work were preliminary, further research is needed to study the exact methylation levels among the population born in the famine years to fully validate this hypothesis. In addition, there is a need to further investigate more possible confounding factors of SCZ, such as smoking and drinking.

## 5. Conclusions

This study is the first to report that the methylation site rs2283291 on *CACNA1C* was associated with SCZ in Chinese males and the GA genotype of rs2283291 increased the risk of SCZ among Chinese males in the overdominant model.

## Figures and Tables

**Table 1:** Baseline characteristics of subjects.

Items	Famine & SCZ (*n* = 225)	Famine & HC (*n* = 248)	Nonfamine & HC (*n* = 267)	Nonfamine & SCZ (*n* = 220)
Age (years)	49.77 (1.13)	49.65 (1.04)	46.90 (1.03)	46.70 (0.91)
Gender (male, *n* (%))	132 (58.67)	101 (40.73)	113 (42.32)	145 (65.91)
Birthplace (urban, *n* (%))	87 (38.67)	150 (60.48)	145 (54.31)	99 (45.00)
Maternal smoking (*n* (%))	22 (9.78)	35 (14.11)	37 (13.86)	25 (11.36)
Maternal drinking (*n* (%))	5 (2.22)	7 (2.82)	5 (1.87)	5 (2.27)
Maternal illness (*n* (%))	13 (5.78)	10 (4.03)	5 (1.87)	6 (2.73)
Education (below six years, *n* (%))	42 (18.67)	35 (14.11)	37 (13.86)	38 (17.27)

SCZ: schizophrenic; HC: healthy control.

**Table 2:** Association analysis of SNPs (rs2283291 and rs4648635) with SCZ as stratified by the famine exposure (*n* (%)).

Genotype/allele	Famine				Nonfamine			
	SCZ (*n* = 225)	HC (*n* = 248)	*χ* ^2^	*P*	SCZ (*n* = 220)	HC (*n* = 267)	*χ* ^2^	*P*
rs2283291								
GG	106 (47.1)	117 (47.2)	2.386	0.303	94 (42.7)	129 (48.3)	3.464	0.177
GA	104 (46.2)	105 (42.3)			107 (48.6)	108 (40.4)		
AA	15 (6.7)	26 (10.5)			19 (8.6)	30 (11.2)		
G	316 (70.2)	339 (68.3)	0.390	0.532	295 (67.0)	366 (68.5)	0.247	0.619
A	134 (29.8)	157 (31.7)			145 (33.0)	168 (31.5)		
rs4648635								
CC	176 (78.2)	198 (79.8)	—	0.915^∗^	185 (84.1)	216 (80.9)	—	0.617^∗^
CT	46 (20.4)	47 (19.0)			33 (15.0)	49 (18.4)		
TT	3 (1.3)	3 (1.2)			2 (0.9)	2 (0.7)		
C	398 (88.4)	443 (89.3)	0.181	0.671	403 (91.6)	481 (90.1)	0.661	0.416
T	52 (11.6)	53 (10.7)			37 (8.4)	53 (9.9)		

SCZ: schizophrenic; HC: healthy control. ^∗^Fisher's exact test.

**Table 3:** Association analysis of SNPs (rs2283291 and rs4648635) with SCZ as stratified by gender (*n* (%)).

Genotype/allele	Total				Male				Female			
	SCZ (*n* = 445)	HC (*n* = 515)	*χ* ^2^	*P*	SCZ (*n* = 277)	HC (*n* = 214)	*χ* ^2^	*P*	SCZ (*n* = 168)	HC (*n* = 301)	*χ* ^2^	*P*
rs2283291												
GG	200 (44.9)	246 (47.8)	5.054	0.080	117 (42.2)	105 (49.1)	8.151	**0.017**	83 (49.4)	141 (46.8)	0.592	0.744
GA	211 (47.4)	213 (41.4)			139 (50.2)	82 (38.3)			72 (42.9)	131 (43.5)		
AA	34 (7.6)	56 (10.9)			21 (7.6)	27 (12.6)			13 (7.7)	29 (9.6)		
G	611 (68.7)	705 (68.4)	0.009	0.923	373 (67.3)	292 (68.2)	0.089	0.766	238 (70.8)	413 (68.6)	0.504	0.478
A	279 (31.3)	325 (31.6)			181 (32.7)	136 (31.8)			98 (29.2)	189 (31.4)		
rs4648635												
CC	361 (81.1)	414 (80.4)	0.173	0.917	224 (80.9)	169 (79.0)	—	0.164^∗^	137 (81.5)	245 (81.4)	—	0.697^∗^
CT	79 (17.8)	96 (18.6)			49 (17.7)	45 (21.0)			30 (17.9)	51 (16.9)		
TT	5 (1.1)	5 (1.0)			4 (1.4)	0 (0.0)			1 (0.6)	5 (1.7)		
C	801 (90.0)	924 (89.7)	0.044	0.833	497 (89.7)	383 (89.5)	0.013	0.909	304 (90.5)	541 (89.9)	0.090	0.765
T	89 (10.0)	106 (10.3)			57 (10.3)	45 (10.5)			32 (9.5)	61 (10.1)		

SCZ: schizophrenic; HC: healthy control. ^∗^Fisher's exact test.

**Table 4:** Association analysis of SNP genotype distributions in SCZ patients and the healthy control group as stratified by the gender.

SNP	Model	Genotype	SCZ vs. HC (male)			SCZ vs. HC (female)		
			OR (95% CI)	*P*	AIC	OR (95% CI)	*P*	AIC
rs2283291	Codominant	GG	1.00 (ref)	0.017	670.4	1.00 (ref)	0.74	617.3
		GA	1.52 (1.04-2.22)			0.93 (0.63-1.39)		
		AA	0.70 (0.37-1.31)			0.76 (0.38-1.55)		
	Dominant	GG	1.00 (ref)	0.13	674.3	1.00 (ref)	0.59	615.6
		GA+AA	1.32 (0.92-1.89)			0.90 (0.62-1.32)		
	Recessive	GG+GA	1.00 (ref)	0.064	673.1	1.00 (ref)	0.49	615.4
		AA	0.57 (0.31-1.04)			0.79 (0.40-1.56)		
	Overdominant	GG+AA	1.00 (ref)	**0.0086**	**669.7**	1.00 (ref)	0.89	615.9
		GA	1.62 (1.13-2.33)			0.97 (0.66-1.42)		

rs4648635	Codominant	CC	1.00 (ref)	0.07	673.2	1.00 (ref)	0.57	616.8
		CT	0.82 (0.52-1.29)			1.05 (0.64-1.73)		
		TT	—			0.36 (0.04-3.09)		
	Dominant	CC	1.00 (ref)	0.6	676.3	1.00 (ref)	0.97	615.9
		CT+TT	0.89 (0.57-1.39)			0.99 (0.61-1.61)		
	Recessive	CC+CT	1.00 (ref)	—	—	1.00 (ref)	0.30	614.8
		TT	—			0.35 (0.04-3.06)		
	Overdominant	CC+TT	1.00 (ref)	0.35	675.7	1.00 (ref)	0.80	615.9
		CT	0.81 (0.51-1.27)			1.07 (0.65-1.75)		

SCZ: schizophrenic; HC: healthy control.

**Table 5:** Crossover analysis of the interactions between rs2283291/rs4648635 and famine factor with SCZ.

Interaction term		SCZ	HC	OR (95% CI)	*P*
rs2283291	Famine				
+	+	119	131	1.247 (0.867, 1.793)	0.235
+	-	126	138	1.253 (0.875, 1.794)	0.218
-	+	106	117	1.243 (0.856, 1.807)	0.253
-	-	94	129	1 (ref)	
rs4648635	Famine				
+	+	49	50	1.144 (0.737, 1.777)	0.548
+	-	35	51	0.801 (0.499, 1.286)	0.358
-	+	176	198	1.038 (0.782, 1.377)	0.797
-	-	185	216	1 (ref)	

SCZ: schizophrenic; HC: healthy control; rs2283291 (+): mutant (GA+AA); rs2283291 (-): wild-type (GG); rs4648635 (+): mutant (CT+TT); rs4648635 (-): wild-type (CC).

## Data Availability

The data used to support the findings of this study are available from the corresponding author upon request.
